# Behavioral and subcortical signatures of musical expertise in Mandarin Chinese speakers

**DOI:** 10.1371/journal.pone.0190793

**Published:** 2018-01-04

**Authors:** Caitlin Dawson, Mari Tervaniemi, Daniel Aalto

**Affiliations:** 1 Cognitive Brain Research Unit, Faculty of Medicine, University of Helsinki, Helsinki, Finland; 2 CICERO Learning Network, Faculty of Educational Sciences, University of Helsinki, Helsinki, Finland; 3 Communication Sciences and Disorders, Faculty of Rehabilitation Medicine, University of Alberta, Edmonton, Canada; 4 Institute for Reconstructive Sciences in Medicine, Misericordia Community Hospital, Edmonton, Canada; Universitat Zurich, SWITZERLAND

## Abstract

Both musical training and native language have been shown to have experience-based plastic effects on auditory processing. However, the combined effects within individuals are unclear. Recent research suggests that musical training and tone language speaking are not clearly additive in their effects on processing of auditory features and that there may be a disconnect between perceptual and neural signatures of auditory feature processing. The literature has only recently begun to investigate the effects of musical expertise on basic auditory processing for different linguistic groups. This work provides a profile of primary auditory feature discrimination for Mandarin speaking musicians and nonmusicians. The musicians showed enhanced perceptual discrimination for both frequency and duration as well as enhanced duration discrimination in a multifeature discrimination task, compared to nonmusicians. However, there were no differences between the groups in duration processing of nonspeech sounds at a subcortical level or in subcortical frequency representation of a nonnative tone contour, for *f*_*o*_ or for the first or second formant region. The results indicate that musical expertise provides a cognitive, but not subcortical, advantage in a population of Mandarin speakers.

## Introduction

The plastic effects of musical training on the brain have gained great interest in the research community [1]. Musical training has been shown to be associated with perceptual benefits in lower frequency discrimination thresholds for pure tones [2,3] and faster and more accurate detection of small pitch changes [4] not only in nonspeech sounds but also in a foreign language [5], compared to nonmusicians. Musicians have shown enhanced mismatch negativity (MMN) to slightly detuned chords, indicating more precise detection of frequency deviations [6]. On a subcortical level, musicians show enhanced phase locking and pitch representation in the frequency following response (FFR) in both musical and speech sounds [7,8], enhanced representation of spectral content which contains vocal emotion [9,10], and enhanced differentiation of speech sounds by encoding of the second formant [11]. The magnitude of brainstem responses to tuned and detuned chords was also related to perceptual differences in pitch discrimination between musicians and nonmusicians, indicating a link between behavioral performance and subcortical plasticity [12].

Auditory plasticity has been shown over short periods of time in schoolchildren participating in both formal and informal musical activities, indicating that experience-based effects of music are not limited to adult professional musicians but that musical experience promotes maturation of the auditory system [13,14,15] and auditory plasticity over the lifespan is sensitive to behavioral needs. The current view is that musical training promotes efficiency through corticofugal tuning which emphasizes features that are trained and/or are useful for the current task demands [16].

Native speakers of tone languages, which encode lexical pitch contrasts, show perceptual benefits for frequency and interval change detection as well as discrimination of nonnative linguistic tone contrasts compared to English speakers, even after training [17,18]. Mandarin speakers have shown stronger pitch representation and smoother pitch tracking to Mandarin tones as well as stronger representation of the second harmonic [19]. Pitch tracking of Mandarin Chinese and Thai speakers to linguistic tone contours was more accurate than that of English speakers, indicating a transfer effect between tone languages [20]. Moreover, pitch representation is enhanced to musical and nonmusical sounds, speech stimuli, and iterated ripple noise, which suggests an effect that is not specific to the speech context [21,22,23].

Effects of musical training and tone language are very similar, and many studies have equivocated them. However, recent attempts to disentangle the effects have shown a much more complex picture. Cooper and Wang [24] separated tone and non-tone speakers and musicians and nonmusicians in both linguistic groups (English and Thai) and taught them a new tone language (Cantonese). They found no clear advantage for tone language learning from the native tone language speaking musicians; rather, English-speaking musicians had the most advantage in learning Cantonese. The Thai speakers experienced tone confusion which impeded their learning of the new Cantonese tone contours, while the musicians in both linguistic groups performed better than the nonmusicians.

Language effects have been shown not only with tone languages but also quantity languages, like Finnish, which encode lexical duration. Previous studies have shown that native speakers exhibit enhanced perceptual, cortical, and duration processing at subcortical level [25,26,27,28]. The interaction of these effects with musical training, however, is more complex, and the effects of musical expertise within linguistic groups are unclear. Enhanced MMNs and perceptual detection for duration deviants was found for Finnish speaking nonmusicians and French speaking musicians, but enhanced MMNs were found for frequency deviants only in French speaking musicians [29]. Likewise, Finnish speakers with greater musical sophistication have shown enhanced perceptual frequency discrimination, but not duration discrimination, and no enhanced subcortical duration discrimination, compared to those with less musical sophistication [30]. These studies indicate a specific effect of native language phonological patterns in the effects of musical expertise within the linguistic group.

Other research has shown an interesting disconnect between perceptual and neural effects when music and language are investigated in combination. Bidelman, Gandour, and Krishnan [31] found enhanced subcortical representation of pitch sequences in both musicians and Chinese speakers but only corresponding perceptual pitch discrimination advantages for the musicians, indicating that cognitive benefits of auditory training may arise only for behaviorally relevant tasks.

On the other hand, Hutka et al. [32] found enhanced perceptual pitch discrimination for both musicians and Cantonese speakers, compared to nonmusicians, but only enhanced MMNs for pitch and timbre deviants in musicians. The authors interpret this as musical training having broader benefits to auditory processing than language, which is more specific. The divergence of results between several studies suggest that music and language may have different mechanisms or effects on plasticity; i.e. they do not appear to be clearly additive.

Moreover, there is a lack of linguistic group control in the language and music literature and little knowledge about the effects of musical expertise within different linguistic groups, particularly tone language speakers. If musical training and native language possibly have different mechanisms or interacting effects, then they must be adequately controlled in future research. This study attempts to contribute to the illumination of the separate effects of musical expertise and native language by investigating the effects of musical expertise on native speakers of a tone language (Mandarin Chinese). It uses both perceptual auditory feature discrimination tasks and brainstem recording designed to spotlight onset and sustained responses for subcortical duration and frequency signatures in order to form a thorough profile of the effects of musical expertise in Mandarin speakers.

## Methods

### Participants

57 native Mandarin Chinese speaking adults aged 18–35 participated in behavioral data collection (21 males, 28 nonmusicians, 29 musicians; Table 1).

**Table 1:** Description of participants: group designation, age, gender, Gold-MSI scores, and primary instruments of musicians.

	Total	Mean age	Gender	Mean Gold-MSI
			**M F**	
**Nonmusicians**	**29**	**21.9**	**9 20**	**54.6**
**Musicians**	**28**	**20.5**	**12 16**	**97.6**
*Piano*	9	***Western*** ***Instruments***		
*Violin*	1			
*Electric keyboard*	1			
*Guitar*	5			
*Electric guitar*	2			
*Drums*	2			
*Saxophone*	1			
*Voice*	2			
*Bamboo flute*	1	***Traditional*** ***Instruments***		
*Yangqin*	1			
*Erhu*	1			
*Guzheng/koto*	2			

55 of them also participated in the auditory brainstem response (ABR) data collection (20 males, 26 nonmusicians, 29 musicians). No participants had any experience with Finnish and spoke primarily Mandarin Chinese at home for the first 15 years of life. Some studies have shown connections between auditory discrimination and intelligence [33,34,35,36], but for practical reasons, it was not possible to conduct large-scale intelligence testing.

Musicians were defined as having more than 6 years of formal musical training and weekly musical practice, and nonmusicians were defined as having fewer than 2 years of musical training and no regular musical hobbies.

Participants were recruited by student telephone phone and email lists within Beijing Normal University and were compensated for their time. They gave written consent according to the Declaration of Helsinki and the ethical review committees of both the University of Helsinki and Beijing Normal University.

### Procedure

The full experiment took 2 hours and all participants completed the brainstem recording first. The recording consisted of two blocks of a passive listening task, counterbalanced between participants in order to avoid any attentional issues that may affect data quality (boredom, movements, etc.). The first block contained two synthesized short sounds (see section: Stimuli) presented at 55 dB sound pressure level (SPL). The second block contained one natural consonant-vowel (CV) speech contour, /*puu*/, extracted from a longer Finnish word /*puuro*/ which means “porridge,” presented at 65 dB SPL. There were a total of 6000 sweeps for each short stimulus (3000 per polarity) and 4000 sweeps for the speech stimulus.

For brainstem recording, a one-channel setup was used with one active channel at Cz online referenced to linked mastoids with a forehead ground at the hairline and four vertical and horizontal electrooculography (EOG) electrodes. A ±30 μV thresholding process was applied for artifact rejection. Data was collected in a shielded room using a Neuroscan SynAmps^2^ Scan 4.5 system with a sampling rate of 20 kHz in AC mode/Gain 2010 and online open filter 10–3000 Hz with 6 dB roll-off. Sound stimuli were presented binaurally with shielded circumaural Sennheiser HD 419 headphones.

The behavioral experiment consisted of four listening tests modified from Kaernbach [37]. Participants listened to sounds with headphones presented from a laptop with sound calibrated to 65dB SPL. There were three adaptive single-feature tasks in which one sound feature was adjusted at a time (intensity, frequency, or duration) in order to find the 75% accuracy threshold for each feature. During each trial, two sounds were played in sequence and the participant was asked to press a key on the laptop to choose which sound was louder, higher, or longer (Intensity Test, Frequency Test, Duration Test, respectively). Correct answers increased the task difficulty by one step and incorrect answers reduced task difficulty by three steps (one-up three-down procedure), to find an accuracy rate of 75%. These tasks took about 10 minutes each. Then, a multifeature task asked again which sound was longer (duration), but all three features were varied randomly. This task took 20 minutes and terminated after 300 trials.

### Stimuli

The first block of the ABR section consisted of two synthesized narrowband gamma-filtered stimuli, one at 162 Hz and on at 216 Hz, both presented at 55dB (SPL). A sawtooth wave of each pitch was narrow band filtered using a fourth order polynomial gammatone filter with centre frequency 3141.56 Hz; then, average intensities were normalized. Each stimulus is about 25ms in length with a 25ms silent buffer before and after the sound for an interstimulus interval (ISI) of about 50ms (the lengths are not actually absolute since the duration of the stimuli depend somewhat on the periodicity of the frequencies). The short stimuli were presented in alternating polarities and randomized.

The second block of the ABR section consisted of one CV syllable, */puu/*, which means “tree,” recorded from an adult female native Finnish speaker and cut from the longer word */puuro/*, which means “porridge.” The tone contour ranges in fundamental frequency (*f*_*o*_) from 169 to 233 Hz and lasts 340ms long with a 20ms silence before and 30ms silence after, presented in a single polarity at 65 dB with a total of 4000 sweeps. Finnish does not have a system of lexical tones as Mandarin does, but instead a lexical duration contrast, in which vowels and consonants have a long and short version, e.g. *tuli*, “fire,” *tuuli*, “wind,” and *tulli*, “customs.” The long vowels are co-signaled by a tone contour which has a slight initial rise followed by a long fall which aids in recognition of duration contrasts. Mandarin has four lexical tones: high level, high rising, low falling-rising, and high falling. Thus, the tone contour used here came from a natural spoken language but represented a totally unfamiliar contour to Mandarin speakers.

The behavioral stimuli were synthesized in the same way as the short nonspeech sounds used for brainstem recording but were longer since they were used for perceptual judgments. The standard sounds were 150ms long, 65 dB, and 162 Hz. The behavioral tasks were created within custom Matlab functions to be within the range of human speech syllables in intensity, frequency, and duration. The three features were either held constant or varied adaptively or randomly, depending on the task. The adaptive tasks automatically terminated after 51 reversals; the multifeature task had 300 trials.

## Analysis

### Psychoacoustic tasks

The behavioral analysis used estimates from a logistic regression model that were fitted to the binary response data to calculate Weber fractions that represent discrimination thresholds for each auditory feature, using the equation *ln(3)/k* where *k* is the GLM estimate. For the duration modulation test, *generalized Weber fractions* use the same calculation and represent the extent to which duration is judged longer, given an increase in each specific feature (intensity, frequency, or duration). Additional effects were calculated: the intensity ratio, which is the (absolute value of the) ratio of generalized Weber fractions for the intensity dimension over the duration dimension and represents the extent to which participants were influenced by variation in intensity when making the duration judgment (a larger ratio corresponds to more influence). The frequency ratio is the same calculation for the influence of frequency on duration judgment, and the duration ratio is the ratio of Weber fractions of duration discrimination from the simple task to the complex task, which represents the difference in performance between the simple and complex tasks (a smaller ratio corresponds to decrement in performance from simple to complex task). It is expected that all participants decrease in performance between the simple and complex task since ignoring distracting features is a more difficult task.

### Subcortical responses

For analysis of the short ABR stimuli, data was preprocessed with band-pass filters at 80Hz and 4000Hz and an artifact rejection threshold of 30 μV and epochs of 15ms prestimulus and 30ms poststimulus. Due to a technical error, it was not possible to separate responses to the two different stimuli; therefore, the results show group grand averages. Wave V peak amplitudes and latencies were extracted with a custom Matlab thresholding algorithm designed to detect peaks within a designated time window as a percentage of total peak size, which is a conservative measure to take higher-amplitude noise into consideration. Wave V is thought to be generated by the inferior colliculus, which is a waystation for corticofugal connections and is an important integration point for incoming afferent and efferent information. The amplitude of wave V indicates precision in the temporal tuning of a population of neurons responding to sound [38]. It has been shown to reflect subcortical experience-based plasticity from auditory training and is affected by learning and language disorders [39,40,41]. It has previously been shown that wave V amplitude reflects enhanced duration processing at a subcortical level associated with quantity language experience [28], so the current study was interested in possible duration processing enhancement at the subcortical level due to musical expertise.

Responses to the speech stimulus were bandpass filtered from 80–1000 Hz. The analysis was mainly concerned with the sustained portion of the response (post-20ms). Waveforms for each subject were averaged before further analysis.

FFR analysis was conducted by means of a sliding window short-term autocorrelation function which allocated 40 ms time bins shifted by 1ms, creating 283 overlapping bins. For the pitch tracking analysis, each bin was autocorrelated (cross-correlated with itself) and the peak autocorrelation value (expressed as a number between 0 and 1, excluding the first lag which is 1) was identified for each bin, representing the periodicity strength of each time bin. Then, these peak values were averaged for each participant to determine the participant's pitch strength over the entire course of the response.

A short-term spectral analysis was also conducted using the same sliding window function. A Fast Fourier Transform (FFT) was applied to the windowed bins (Hanning window, bins zero-padded to 1 second to increase spectral resolution). From this, it was possible to extract the *f*_*o*_ contour from the spectrogram by identifying the frequency which shows the peak magnitude for each time bin. Thus, this is the measure of pitch tracking in terms of frequency. These peak magnitude frequencies per subject were then cross correlated with the stimulus itself (which has undergone the same short-term FFT process) to obtain the FFT pitch tracking measure (expressed as a cross correlation coefficient between 0 and 1) per participant.

### Musical expertise

For measures of musical expertise used in correlations, the current study uses the generalized score of the self-report questionnaire from the Goldsmiths Musical Sophistication Index (Gold-MSI) [42]. As a full evaluation it consists of the self-report questionnaire and a battery of listening tests including melodic memory, beat perception, and sound similarity. The self-report questionnaire alone has been validated using objective listening tests and is an effective measure of musical ability [43]. The self-report inventory scores participants along five factors of musical engagement: active engagement, perceptual abilities, musical training, singing abilities, and emotional engagement. These factors are weighted together to create the generalized musical sophistication score. The Gold-MSI is equally useful for evaluating the musical sophistication of people who are highly formally trained, untrained, or have casual musical experience.

### Statistical analysis

Since the distributions were not normal, nonparametric methods were used. A series of Mann-Whitney-Wilcoxon tests were run to compare the results of each test between music groups and Bonferroni corrected for multiple comparisons within effect type.

An additional comparison was done of pitch tracking in responses to the speech stimulus with a restricted frequency window of 100Hz around the first and second formants. A further analysis correlated Gold-MSI general sophistication scores with all of the previous effects: behavioral single-feature frequency, intensity, and duration discrimination, multifeature duration discrimination, frequency ratio, duration ratio, intensity ratio, wave V amplitude and latency, autocorrelation pitch tracking, FFT pitch tracking for *f*_*o*_, *F*_*1*_, and *F*_*2*_.

## Results

### Perceptual effects

Musicians showed enhanced single-feature discrimination for both frequency and duration and for duration in the complex task compared to the non-musicians (for descriptives, see Table 2). They also showed a trending difference in single-feature intensity discrimination and frequency ratio, which did not reach significance at the corrected level (Table 3).

**Table 2:** Mean and standard deviation of Weber fractions for musicians and nonmusicians for each perceptual variable of interest.

Weber fractions	Mean		Std dev	
	Musicians	Nonmusicians	Musicians	Nonmusicians
**Frequency**	0.5	7.42	0.76	18.4
**Duration**	0.025	0.066	0.016	0.13
**Intensity**	1.14	1.59	0.67	1.06
**Complex duration**	0.031	0.057	0.026	0.056
**Frequency ratio**	156.28	-1259.4	631.45	6596.13
**Intensity ratio**	1474.97	233.69	6280.8	965.24
**Duration ratio**	0.86	1.14	0.33	1.17

**Table 3:** Mann-Whitney-Wilcoxon test for perceptual effects between musicians and nonmusicians.

Effect	W value	P value
**Simple frequency**	145	0.000087*
**Simple duration**	199	0.0022*
**Simple intensity**	269	0.067
**Complex duration**	216	0.0035*
**Frequency ratio**	526	0.0279
**Intensity ratio**	479	0.16
**Duration ratio**	338	0.51

Bonferroni-corrected alpha level at 0.007.

* indicates significance at the Bonferroni-corrected level.

### Subcortical effects

**Fig 1:**
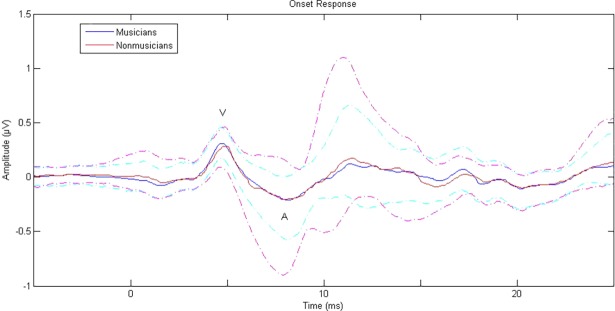
Onset response of musicians (blue, dark) and nonmusicians (red, light) to synthesized nonspeech sounds showing V-A complex. Dashed lines represent one standard deviation.

**Fig 2:**
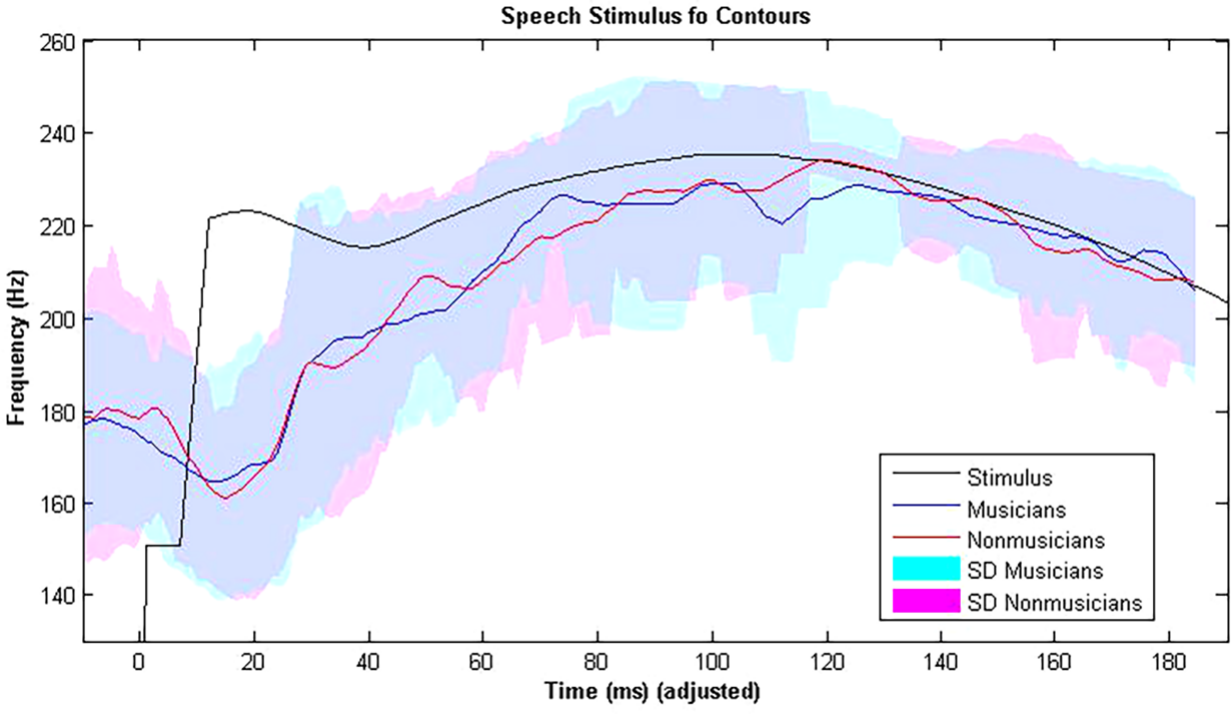
f_o_ contours of musicians (blue, dark) and nonmusicians (red, light) to a natural speech sound /puu/. The grey line shows the contour of the original stimulus, and dashed lines represent one standard deviation.

There were no differences between musicians and nonmusicians for either peak amplitude or latency of wave V in onset responses to the short nonspeech stimuli (Fig 1), nor for either autocorrelation pitch tracking or FFT pitch tracking of fundamental frequency in responses to the speech stimulus (Fig 2, Table 4).

**Table 4:** *Mann-Whitney-Wilcoxon test for subcortical effects between musicians and* nonmusicians.

Effect	W value	P value
**Autocorrelation pitch tracking**	377	0.99
**FFT pitch tracking *f*_*o*_**	402.5	0.69
**Wave V peak amplitude**	334	0.68
**Wave V peak latency**	312	1

Bonferroni-corrected alpha level at 0.0125.

* indicates significance at the Bonferroni-corrected level.

### Further analysis

#### Formant pitch tracking

The FFT pitch tracking sliding window algorithm was run again on 100 Hz windows around the average first (397–497) and second (700–800) formant frequencies as identified by Praat. The sliding window was also run on the original speech stimulus with the same restrictions and the results were cross correlated. Pitch tracking of the formants was also not significantly different between musicians and nonmusicians (Table 5).

**Table 5:** Mann-Whitney-Wilcoxon test for first and second formant pitch tracking between musicians and nonmusicians.

Effect	W value	P value
**FFT pitch tracking *F*_*1*_**	346.5	0.77
**FFT pitch tracking *F*_*2*_**	362.5	0.99

Bonferroni-corrected alpha level at 0.025.

* indicates significance at the Bonferroni-corrected level.

#### Correlations with musical sophistication

A further analysis determined whether the results would be different with another measure of musical expertise, namely, the Gold-MSI general musical sophistication score. This score takes into consideration formal musical training but also factors which are unrelated to training and which may be due to aptitude or social or environmental conditions. All previously used perceptual and neural measures were correlated with the general musical sophistication index score, and the results mirror those of the music groups although some of the trends do not reach the corrected significance level (Tables 6 and 7). Moreover, the group music score means were significantly different, indicating that the participants were accurately assigned to musician and nonmusician groups (W = 812, p = 9.52 x10^-11^). The difference is likely due to the fact that the two measures of musical expertise emphasize slightly different factors.

**Table 6:** Correlations between Gold-MSI generalized musical sophistication score and perceptual effects.

Correlations	S	*rho*	P value
**Music x frequency**	39652	-0.51	.000077*
**Music x duration**	37090	-0.34	0.012
**Music x intensity**	31945	-0.15	0.27
**Music x complex duration**	37825	-0.29	0.029
**Music x frequency ratio**	21020	0.28	0.036
**Music x intensity ratio**	26078	0.11	0.43
**Music x duration ratio**	30894	-0.11	0.41

Bonferroni-corrected alpha level at 0.007.

* indicates significance at the Bonferroni-corrected level.

**Table 7:** Correlations between Gold-MSI generalized musical sophistication score and subcortical effect.

Correlations	S	*rho*	P value
**Music x Autocorrelation pitch tracking**	27548	0.0062	0.96
**Music x FFT pitch tracking *f*_*o*_**	25003	0.098	0.48
**Music x FFT pitch tracking *F*_*1*_**	23952	0.087	0.53
**Music x FFT pitch tracking *F*_*2*_**	25773	0.018	0.90
**Music x Wave V peak amplitude**	19513	0.063	0.66
**Music x Wave V peak latency**	17492	0.16	0.27

Bonferroni-corrected alpha level at 0.008

* indicates significance at the Bonferroni-corrected level.

## Discussion

This work investigated the basic perceptual and subcortical auditory profiles of Mandarin speaking musicians and nonmusicians. Mandarin speaking musicians showed more accurate single-feature discrimination for both frequency and duration and a stronger influence of frequency on duration discrimination in a complex auditory environment. No subcortical effects were found.

### Perceptual effects

There was no effect of duration ratio, which means that there was no group-based difference in the relationship between the single-feature duration task and the multifeature duration task. In general, participants decline in accuracy between the simple and complex tasks due to the increase in processing load from the addition of distracting features. It might be expected that musicians would perform better in the complex task (showing less decrement in performance) than nonmusicians due to their superior processing skills. However, it may also be argued that enhancement in processing of low level single features could lead to an overall increase in system efficiency, which promotes integration of low level features. This appears to be what happened in a population of musically diverse Finnish speakers, whose linguistically driven enhancement for duration processing was more degraded in the complex task for those with higher levels of musical sophistication [30]. Here, there was no difference in degradation of duration discrimination for Mandarin speakers with the addition of distracting features between musicians and nonmusicians. In fact, the musicians showed significantly more accurate duration discrimination in the complex task compared to the nonmusicians. In other words, both the Mandarin speaking musicians and nonmusicians showed a similar extent of degradation between the simple and complex task, but the musicians had an overall more accurate duration discrimination within both tasks.

There was a nonsignificant trend (at corrected level) of frequency ratio. Previous studies have found that Mandarin speakers are less affected by frequency when making duration judgments than quantity language speakers (Finnish and Estonian), with both the most accurate duration discrimination and the most influence of frequency on duration discrimination occurring for Finns [44]. The positive correlation indicates that the more musically sophisticated participants were *more* affected by frequency in their duration judgments than the less musically sophisticated. Although counterintuitive, this indicates an enhanced efficiency in the auditory system since psychoacoustically, frequency contributes to perceived duration [45,46]. By integrating features which are perceptually bound, musicians process sound more efficiently in real-world acoustic environments like music performance.

### Subcortical effects

Both groups showed high variability in amplitude of the onset response. Both groups accurately followed the speech stimulus tone contour, however, FFT pitch tracking for both musicians and nonmusicians, while giving generally high cross correlation values, was similarly highly variable and contained octave jumps. It is likely that since the participants were all healthy adult native Mandarin speakers, there was a ceiling effect for subcortical frequency processing due to linguistic expertise.

The speech stimulus was chosen to represent a nonnative tone contour from a natural language. It is possible that the Mandarin speakers did not process the stimulus as linguistic, and/or that the musicians processed it as musical, which would activate perceptual benefits from cognitively identifying the task demands in a musical context. Previous research has shown top-down effects of language or music on categorization (and further pitch processing) of sounds which are similar to natural language tone contours or musical notes [47,48]. It may be necessary to direct participants’ “listening mode” with stimuli that could be ambiguously interpreted to be linguistic or musical. Additionally, further investigations could use a wider range of similar natural speech, musical, and speech like stimuli, such as instrumental and vocal contours, synthesized contours without phonemes, and iterated ripple noise in order to determine the effect of top-down organization of auditory domains.

### Musical expertise

The correlational analysis with Gold-MSI scores showed the same pattern of effects as the cross-sectional analysis, which was expected since the group means were significantly different. However, the distribution of scores was not bimodal, as would be expected from groups which did not overlap in level of musical training (fewer than 2 years/6 or more years). The Gold-MSI is likely capturing additional features that are not directly associated with formal musical training and which may have a weak effect on the results.

Participants indicated their main instrument on the Gold-MSI (Table 1). Of the 28 musicians, 21 indicated Western instruments, 5 indicated traditional Chinese instruments, and 2 indicated voice. The traditional instruments included *guzheng* (Chinese zither), *koto* (Japanese instrument similar to the *guzheng*), *yangqin* (a hammered dulcimer), *erhu* (a two-stringed fiddle), and bamboo flute. Previous research has shown that there are differences in auditory feature processing between different kinds of instrumentalists and musical styles [49,50,51]. Here, it is possible that different styles or cultures of music training could emphasize different auditory features enough to influence the results. Unfortunately, the Western and traditional groups here were too different in number to compare in a statistically meaningful way. However, musical culture remains an interesting question for the future and could be investigated by focusing on style of musical expertise as a design factor.

### Limitations

As mentioned above, it was not possible to statistically compare musicians trained in traditional or Western musical styles. It would be of particular interest to compare musicians trained in different tonal systems or on fixed- and movable pitch instruments or vocalists since regular practice of a tonal system with smaller or larger frequency differences between notes may influence discrimination patterns.

One of the main limitations of this work is the lack of a multifeature frequency discrimination task. In the future, some of these questions could be addressed by a more complete set of perceptual tasks, especially since the Mandarin speakers show music-based effects for both frequency and duration.

Some recent research has indicated genetic factors in auditory feature processing and musical aptitude heritability [52,53,54,55,56]. Future studies should consider the impact of genetic differences across major linguistic groups and the effect that difference may have in comparing auditory processing between the groups.

## Conclusions

Knowledge about early auditory processing plasticity is becoming more granular and effects specific to certain sound environments are becoming clearer. Future investigations must take into consideration the differences between language environments and musical environments in their effects in tuning the auditory system. Additionally, in order to gain a more complete picture of the plasticity of the auditory system, musicality evaluations should be carefully considered as well as other factors like genetics/aptitude, socio-cultural differences in music attitudes, and behavioral task demands. Musical expertise appears to confer mainly perceptual advantages *within* linguistic groups. The transfer between language and music effects happen on an early level of processing, but responses are still modulated by behavioral goals which drive efferent connections as well as a holistic pressure to efficiency in the full system.

## Supporting information

S1 Table Data values. Data values for each participant used in statistical analyses and demographic information.
(XLSX)
